# Anti-inflammatory effect of the sclerotium of *Lignosus rhinocerotis* (Cooke) Ryvarden, the Tiger Milk mushroom

**DOI:** 10.1186/1472-6882-14-359

**Published:** 2014-09-25

**Authors:** Sook Shien Lee, Nget Hong Tan, Shin Yee Fung, Si Mui Sim, Chon Seng Tan, Szu Ting Ng

**Affiliations:** Department of Molecular Medicine, Faculty of Medicine, University of Malaya, 50603 Kuala Lumpur, Malaysia; Department of Pharmacology, Faculty of Medicine, University of Malaya, 50603 Kuala Lumpur, Malaysia; Biotechnology Research Centre, MARDI, P.O.Box 12301, 50744 Kuala Lumpur, Malaysia; Ligno Biotech Sdn Bhd, Taman Perindustrian Balakong Jaya 2, Selangor, 43300 Balakong Jaya, Malaysia

**Keywords:** *Lignosus rhinocerotis*, Sclerotium, Anti-inflammatory, Carrageenan-induced paw edema, Cotton pellet induced granuloma

## Abstract

**Background:**

The sclerotium of *Lignosus rhinocerotis* (Cooke) Ryvarden (Tiger Milk mushroom) is used as a traditional medicine to relieve cough, asthma and chronic hepatitis. The traditional uses of the sclerotium are presumably related to its anti-inflammatory effect. The present study was carried out to evaluate the anti-inflammatory activity of the sclerotial powder of *L. rhinocerotis* (Cooke) Ryvarden (Tiger Milk mushroom) cultivar TM02.

**Methods:**

The anti-acute inflammatory activity of the sclerotial powder of *L. rhinocerotis* cultivar TM02 was investigated using carrageenan-induced paw edema test while the inhibition of transudative and proliferative phases of chronic inflammation were studied by cotton pellet induced granuloma model. *Sprague Dawley* rats were used in both studies. The anti-inflammatory activity was also measured by inhibition of lipopolysaccharide induced TNF-alpha production in RAW 264.7 macrophage cells.

**Results:**

Cold water extract (CWE), hot water extract (HWE) and methanol extract (ME) of the sclerotial powder of *L. rhinocerotis* cultivar TM02 possessed anti-acute inflammatory activity as was measured by carrageenan-induced paw edema test, with CWE being the most potent. The acute anti-inflammatory activity of the cold water extract (CWE) was mainly contributed by its high molecular weight (HMW) fraction isolated by Sephadex G50 gel filtration chromatography. Its protein component was very potent in the inhibition of TNF-alpha production with an IC_50_ of 0.76 μg/ml. CWE at 200 mg/kg did not inhibit transudative and proliferative phase of chronic inflammation as shown by using the cotton pellet induced granuloma model.

**Conclusions:**

Our results suggested that most of the bioactive substance(s) contributed to the acute-inflammatory activity of the sclerotial powder of *L. rhinocerotis* cultivar TM02 appear to be in the CWE, particularly its HMW fraction. The anti-inflammatory activity of CWE was mainly contributed by the protein component of the high molecular weight (HMW) fraction and it exhibited strong inhibitory effect on TNF-alpha production but the possibility of synergistic effect between HMW, MMW and LMW fractions cannot be excluded. Future studies will provide new insights into the anti-inflammatory activity of the sclerotial powder.

## Background

Inflammation is a normal protective response to tissue injury caused by physical trauma (cut, burn or bruise), noxious chemicals, microbiologic agents or even autoimmune disease [1]. Excessive inflammation may, however, lead to tissue injury and when severe, cause physiological decompensation, organ dysfunction and death cascade [2].

Non-steroidal anti-inflammatory drugs (NSAIDS) which are effective for the treatment of inflammation are among the most widely used drugs in the world [3]. However, NSAIDs are associated with several side effects, especially gastrointestinal toxicity including ulcerations, erosions, life-threatening perforations or severe hemorrhage [4]. Therefore, there is a need to search for alternative anti-inflammatory agent with reduced side effects.

*Lignosus rhinocerotis* (Cooke) Ryvarden (Tiger Milk mushroom, synonym: *Lignosus rhinocerus*), is an important medicinal mushroom in Southeast Asia and China. In Malaysia, it is also known as ‘*cendawan susu rimau*’ and is the most popular medicinal mushroom used by the indigenous communities of Peninsular Malaysia to relieve fever, cough, asthma, cancer, food poisoning and as a general tonic [5, 6]. In China, the sclerotium of the mushroom is an expensive traditional medicine used for the treatment of liver cancer, chronic hepatitis and gastric ulcers [7]. The sclerotium of *L. rhinocerotis* is the part with medicinal value. Unfortunately, the existence of this mushroom in the jungle is always solitary and this makes collection of large quantity of the mushroom’s sclerotia a difficult task. Recently, Tan [8] reported a successful cultivation of the mushroom in agar, solid and spawn medium with good yield, thus making it possible to fully exploit its medicinal and functional benefits. To date, anti-inflammatory activity of *L. rhinocerotis* has not been investigated. In the present study, we report the investigation of the *in vivo* anti-inflammatory activity of the extracts of sclerotial powder of the mushroom using a rat model as well as the *in vitro* inhibitory activity on lipopolysaccharide induced TNF-alpha production in macrophage cells.

## Methods

### Preparation of the sclerotial powder of *L. rhinocerotis*cultivar TM02

The sclerotia of *L. rhinocerotis* cultivar TM02 was provided by Ligno Biotech Sdn. Bhd. (Selangor, Malaysia) and were identified by their internal transcribed spacer (ITS) regions of the ribosomal DNA [9]. Sclerotia were freeze-dried and milled into powder using 0.2 mm sieve. The powder is light brown, dry fluffy with milk-like taste.

### Extraction from the sclerotial powder of *L. rhinocerotis*cultivar TM02

Cold water extract (CWE) was prepared by continual stirring of the sclerotial powder for 24 h in distilled water at 4°C. The extract was termed CWE TM02. Hot water extraction of the sclerotial powder was performed using an oil bath at 95°C to 100°C for 2 h. The extract was termed HWE TM02. Methanol extraction was carried out by continual stirring of the powder in methanol at 25°C for 24 h. The extract was termed ME TM02. All extractions were performed with sample to solvent ratio of 1 g: 20 ml. After each extraction, the mixture was centrifuged at 8000× g for 30 min. The supernatant was filtered to remove insoluble materials. The aqueous extracts were freeze-dried while the methanol extract of the sclerotial powder was evaporated to dryness using at rotary evaporator at 37°C.

### Determination of total carbohydrate and protein contents of the sclerotial extracts of *L. rhinocerotis*cultivar TM02

Total protein content was determined according to Bradford method [10]. Bovine serum albumin was used as a standard. Total carbohydrate content was determined by phenol sulphuric acid method according to Dubois et al. [11]. Glucose standard curve was prepared by using D-glucose.

### Fractionation of CWE of the sclerotial powder of *L. rhinocerotis*

CWE TM02 was fractionated using Sephadex G-50 (Sigma-Aldrich, USA) Superfine column (v = 112 ml) and eluted with 0.05 M ammonium acetate buffer at 0.5 ml/min. High molecular weight (HMW) fraction appeared at void volume (mol. wt. >10 kDa dextran or 30 kDa protein) while low molecular weight (LMW) fraction emerged at bed volume. Medium molecular weight (MMW) fraction appeared between void and bed volume. Protein and carbohydrate distribution of each fraction and the pooled fractions (high, medium and low molecular weight fractions) were determined by Bradford and phenol sulphuric acid method, respectively, as described earlier.

### Animals

Six male *Sprague Dawley* (SD) rats of 9 weeks old (240-270 g) were used for each treatment of carrageenan induced paw edema test while 8 weeks old (170-200 g) were used for cotton pellet induced granuloma test. The animals were supplied by Chenur Supplier (Selangor, Malaysia). The animals were kept under standard conditions (temperature at 22 ± 2°C, 12 h light, 12 h dark), and given food and water *ad libitum*. Animals were acclimatised before use. The animals were handled according to the CIOMS guidelines [12]. Experimental protocols reported in this study were approved by Institutional Animal Care and Use Committee, University of Malaya (UM IACUC-Ethics reference no. PM/16/11/2010/0812/FSY (R)).

### Carrageenan induced paw edema study

Carrageenan induced paw edema method was carried out according to Arawwawala et al. [13], with modifications. CWE TM02 (25, 50, 100 or 200 mg/kg), HMW, MMW and LMW fractions of the CWE (53, 35 and 112 mg/kg, respectively), HWE TM02 (200 mg/kg), ME TM02 (200 mg/kg), reference drug (indomethacin at 10 mg/kg) or control group (6% sodium bicarbonate in saline) was injected intraperitoneally into the rats (2 ml/kg for each rat). The dosages used for HMW, MMW and LMW fractions were calculated based on the fractionated yield of each fraction from 200 mg/kg of CWE. All extracts and the reference drug were prepared in 6% sodium bicarbonate in saline. Intraperitoneal injection was carried out one hour before the carrageenan treatment. Edema was induced by a sub-plantar injection of 1.5% carrageenan in saline (0.2 ml) into the footpad of the right hind paw of all animals. The right hind paw volumes of these rats were measured using a plethysmometer (IITC INC., USA) at hourly intervals up to 5 h. Paw edema volume was calculated by measuring paw volume increment: (V_t_ - V_0_). V_t_ is the average volume of the right hind paw at each time interval after carrageenan injection while V_0_ is the average volume of the right hind paw before carrageenan injection. The percentage inhibition of edema volume at each hour compared to the control group was calculated using the following formula:


Where A= (V_t_ − V_0_)_control_; B= (V_t_ − V_0_)_treatment_

The median inhibitory dose (ID_50_) for the inhibition of inflammation at 3^rd^ and 4^th^ h (peak edema) after inflammation induction was calculated.

### Cotton pellet induced granuloma study

The inhibition effect of CWE on granuloma formation in rats was examined as described by Barua et al. [14]. The rats (n = 6/group) were anesthetised with ketamine (45 mg/kg) and xylazine (4.5 mg/kg) and shaved on their backs. An incision was made on the back followed by subcutaneously implantation of sterilised cotton pellets weighing 20 ± 1 mg. The animals were fed orally with CWE at 200 mg/kg from day 1 to 7. Control group received 6% sodium bicarbonate in saline at 10 ml/kg. Indomethacin group (1 mg/kg) was fed orally every 2 days. On the 8^th^ day, the animals were euthanised using an overdose of carbon dioxide. The cotton pellets surrounded by granulomatous tissue were removed and made free from extraneous tissues. The wet pellets were weighed and dried at 60°C overnight to a constant weight. The mean dry weight of granulomatous tissue (after subtracting the weight of cotton pellet) was determined. The percentage inhibition was calculated by comparing the mean dry and wet weight of the treated group with the control group.

### Isolation of the protein and non-protein components (P-HMW and NP-HMW) of the HMW fraction

The proteins of HMW fraction (P-HMW) were precipitated with ammonium sulphate, which was added gradually to the HMW fraction at 4°C until 100% saturation was reached. After stirring for 1 h, the solution was centrifuged to collect the precipitated proteins. The supernatant was desalted by Sephadex G-50 (Sigma-Aldrich, USA) chromatography (column size v = 228.3 ml) and eluted with 0.05 M ammonium acetate buffer at 2 ml/min. The fractions collected were then freeze-dried. The sample was termed non-protein component of the HMW fraction (NP-HMW). The protein pellet was dissolved in MiliQ water and desalted with Vivaspin 15R (5000 MWCO), and was termed protein component of the HMW fraction (P-HMW). The protein and carbohydrate contents of P-HMW and NP-HMW were determined by Bradford and phenol sulphuric acid method, respectively, as described earlier.

### Determination of the glucan content of the HMW fraction and its protein and non-protein components isolated by ammonium sulfate precipitation

The glucan content of HMW fraction as well as its protein and non-protein components was determined by Mushroom and Yeast Glucan Assay Kit (Megazyme International Ireland Ltd, Bray Co, Wicklow, Ireland) according to manufacturer’s instructions. Briefly, total glucan plus D-glucose in oligosaccharides, sucrose and free D-glucose were solubilised in concentrated hydrochloric acid (37%) and hydrolysed by 1.3 M HCl at 100°C for 2 h. 2 M KOH was then added to neutralise the hydrolysates. Hydrolysis to D-glucose was completed by incubation with a mixture of highly purified exo-1,3-β-glucanase and β-glucosidase. The hydrolysates were then incubated with glucose oxidase/peroxidase mixture (GOPOD) reagent. Absorbance was measured at 510 nm against the GOPOD reagent blank and compared to D-glucose standard to calculate percentage of total glucan plus oligomers etc. Alpha glucan plus D-glucose in sucrose and free D-glucose was solubilised and hydrolysed in 2 M KOH and amyloglucosidase (1630 U/ml) plus invertase (500 U/mL). The hydrolysates were incubated with GOPOD reagent. Absorbance was measured at 510 nm against GOPOD reagent blank and compared to D-glucose standard to calculate percentage of alpha glucan plus oligomers etc. Percentage of beta glucan content was obtained by subtracting percentage of total glucan plus oligomers etc. and alpha glucan plus oligomers.

### Cell culture for studies of inhibition of TNF alpha production

The murine macrophage cell line RAW 264.7 (ATCC® TIB-71™) was grown in DMEM (ATCC) supplemented with 10% heat inactivated FBS, 100 U/mL penicillin, and 100 μg/mL streptomycin, at 37°C in a humidified 5% CO_2_ incubator. To examine cell viability, the cells (seeded at 2 × 10^5^ cells/well in 96-well plate) was pre-treated with the respective extracts for 1 h and then stimulated with 0.10 μg/ml LPS for 24 hours. Medium was then removed and 100 μl of 0.5 mg/ml MTT was added to each well and incubated for 1.5 h at 37°C in the dark. After removing the supernatant, 100 μl DMSO was added to the cells to dissolve the formazan crystals. The absorbance of each group was measured by a microplate reader at 570 nm. Absorbance of treatment groups was normalized to the LPS-treated control, and expressed as percentage viability.

### Measurement of inhibition of TNF-alpha production

RAW 264.7 macrophages were cultured in 96-well plate with CWE, HMW, MMW, LMW and NP-HMW at 12.50, 25.00, 50.00, 75.00 and 100.00 μg/ml, and P-HMW at 0.10, 0.25, 0.50, 0.75, 1.00, 1.25, 1.50, 2.00 and 2.50 μg/ml for 1 h, and then incubated with 0.10 μg/mL LPS for 24 h. Supernatants were obtained and frozen at −80°C until analysis. TNF-alpha in the culture medium was determined by TNF-α mouse ELISA kit (Novex®) according to the manufacturer’s recommended protocols. The ELISA plates were read using a microplate reader at 450 nm. The result was expressed in percentage of inhibition of TNF-alpha production of the various treatment groups as compared to LPS stimulated group without pre-treatment, and IC_50_ values were then calculated.

### Statistical analysis

All data were expressed as mean ± S.E.M. and analysed using One-way Analysis of Variance (ANOVA). Statistical differences between the means of control and treatment groups were determined using Dunnett’s t (two-sided) test. In case of variance heterogeneity, Dunnett’s T3 test was used. The homogeneity of variances was calculated using Levene statistics. Statistical differences between results on 200 mg/kg CWE TM02 and ME TM02 as well as HWE TM02 were analysed using Independent Samples t-test. Results were considered significant at *p* < 0.05.

## Results

### Determination of total carbohydrate and protein content of the various sclerotial extracts

The carbohydrate content of CWE, HWE and ME were determined to be 68.4%, 92.2% and 12.8% by dry weight (w/w), respectively, and the protein content were found to be 2.0%, 0.2% and 0.2% by dry weight (w/w), respectively.

### Extraction Yield of CWE TM02, HWE TM02 and ME TM02

The extraction yield of CWE, HWE and ME were 20%, 37% and 5% by dry weight (w/w), respectively.

### Fractionation of CWE

**Figure 1:**
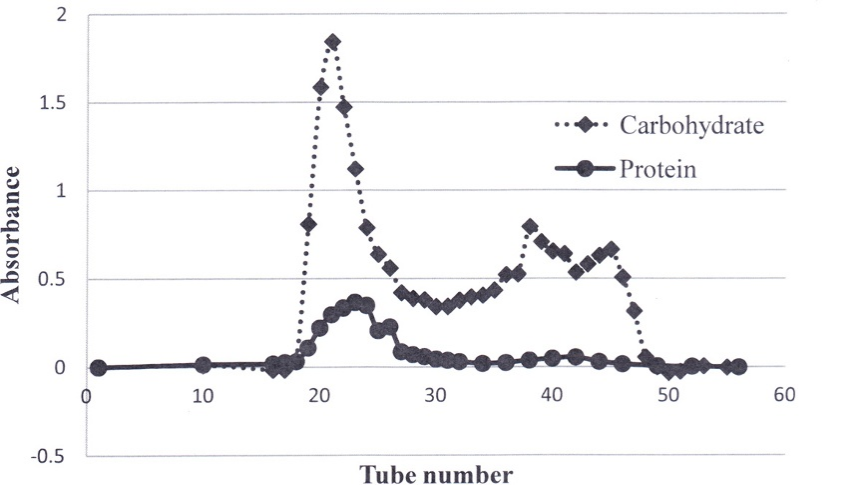
**Sephadex G-50 superfine gel filtration chromatography of the cold water extract (CWE).** The cold water extract (CWE, 500 mg dissolved in 5.6 ml milliQ water) was loaded to the column (v = 112 ml) and 3 ml per fraction were collected into each test tube.

CWE was separated into HMW, MMW and LMW fractions by Sephadex G-50 gel filtration chromatography (Figure 1). The HMW fraction consisted of 4% protein and 79% carbohydrate by dry weight (w/w). The MMW fraction consisted of 1% protein and 54% carbohydrate by dry weight (w/w), while the LMW fraction contained 0.3% of protein and 24% carbohydrate by dry weight (w/w).

### Carrageenan induced paw edema study

CWE was found to reduce carrageenan induced paw edema in SD rats at all doses employed (25, 50, 100 and 200 mg/kg) after 1–5 h of carrageenan injection (Table 1). CWE at 50, 100 and 200 mg/kg displayed significant inhibition of paw edema as compared to control group from 2^nd^ to 5^th^ h post carrageenan injection (p < 0.05). A significant inhibition of paw edema formation was observed in the rat treated with CWE at 200 mg/kg at first hour post carrageenan injection. Paw edema inhibition in dose dependent manner was observed in rat treated with CWE at all doses employed after 2–5 h of carrageenan injection.

**Table 1:** **Effects of sclerotial extracts of** ***L. rhinocerotis*** **cultivar TM02 on carrageenan induced paw edema in rats**

Group	Dose (mg/kg body weight)	Paw edema volume (ml)				
		1 ^st^ hour	2 ^nd^ hour	3 ^rd^ hour	4 ^th^ hour	5 ^th^ hour
**Control**		0.28 ± 0.02	0.76 ± 0.04	0.85 ± 0.03	0.90 ± 0.04	0.86 ± 0.04
**CWE TM02**	**25**	0.25 ± 0.02 (12)	0.52 ± 0.04* (32)	0.70 ± 0.03 (18)	0.70 ± 0.03* (22)	0.65 ± 0.04* (25)
	**50**	0.18 ± 0.02 (36)	0.40 ± 0.07* (48)	0.50 ± 0.06* (42)	0.51 ± 0.05* (44)	0.46 ± 0.05* (47)
	**100**	0.18 ± 0.01 (35)	0.22 ± 0.04* (71)	0.33 ± 0.04* (62)	0.41 ± 0.03* (55)	0.35 ± 0.02* (60)
	**200**	0.11 ± 0.03* (61)	0.13 ± 0.03* (83)	0.10 ± 0.03* (88)	0.11 ± 0.03* (88)	0.10 ± 0.03* (89)
**HMW**	53**	0.11 ± 0.02* (63)	0.12 ± 0.02* (84)	0.12 ± 0.02* (86)	0.09 ± 0.02* (90)	0.08 ± 0.02* (91)
**MMW**	35**	0.16 ± 0.03* (44)	0.30 ± 0.04* (61)	0.35 ± 0.05* (65)	0.51 ± 0.05* (43)	0.44 ± 0.03* (49)
**LMW**	112**	0.23 ± 0.03 (20)	0.46 ± 0.05* (39)	0.51 ± 0.05* (41)	0.51 ± 0.04* (43)	0.47 ± 0.04* (46)
**ME TM02**	**200**	0.24 ± 0.05 (15)	0.68 ± 0.04 (11)	0.78 ± 0.04 (9.0)	0.73 ± 0.04* (19)	0.69 ± 0.03* (20)
**HWE TM02**	**200**	0.20 ± 0.03 (31)	0.41 ± 0.06* (46)	0.50 ± 0.05* (42)	0.49 ± 0.04* (46)	0.47 ± 0.03* (45)
**Indomethacin**	**10**	0.18 ± 0.03 (35)	0.23 ± 0.03* (69)	0.26 ± 0.03* (69)	0.37 ± 0.03* (59)	0.34 ± 0.03*(57)

Each value is mean ± S.E.M of six rats. *p < 0.05, compared with control using Dunnett’s t (two-sided) test. Values given in parentheses represent the percentage inhibition of edema volume at each hour as compared to the control group. **Amount isolated from 200 mg/kg of CWE TM02 using sephadex G-50 gel filtration chromatography. CWE-cold water extract; HWE-hot water extract; ME-methanol extract; HMW-high molecular weight fraction; MMW-medium molecular weight fraction; LMW-low molecular weight fraction.

Paw edema of SD rat treated with CWE reached maximal size at 3^rd^ to 4^th^ h post carrageenan injection. The median inhibitory doses (ID_50_) of the CWE for the suppression of paw edema at the 3^rd^ and 4^th^ h were both 88 mg/kg. CWE at 200 mg/kg showed paw edema inhibition which was higher than inhibition caused by the standard drug indomethacin at 10 mg/kg, after 1–5 h of carrageenan injection. HMW fraction of CWE (53 mg/kg) showed inhibitory effect to paw edema formation in SD rats which was higher than MMW (35 mg/kg) and LMW (112 mg/kg) fractions of the same extract.

HWE and ME at 200 mg/kg displayed paw edema inhibitory activity which was significantly lower (Independent sample t-test, *p* < 0.05) than CWE at the same dosage (after 1–5 h of carrageenan injection), with ME TM02 being the least potent.

### Cotton pellet induced granuloma study

CWE at 200 mg/kg did not significant cause suppression of the wet and dry weight of granuloma as compared to the control group (Table 2). However, the wet and dry weight of granuloma from rats treated with 1 mg/kg indomethacin for every 2 days were significantly reduced by 20.8% and 20.3%, respectively.

**Table 2:** **Effect of the cold water extract (CWE) and indomethacin on cotton pellet induced granuloma in rats**

Treatment	Dose (mg/kg)	Granulomatous tissue	
		Wet weight (mg)	Dry weight (mg)
**6% sodium bicarbonate (control)**	-	281.0 ± 13.8	39.6 ± 1.4
**CWE TM02**	200	281.2 ± 13.9	40.2 ± 2.4
**Indomethacin**	1	222.7 ± 14.3* (20.8%)	31.5 ± 2.6* (20.3%)

Values are mean ± S.E.M. Values given in parentheses represent percentage of inhibition. *Significant difference with control (ANOVA, Dunnett’s t (two-sided) test, *p* < 0.05).

### The protein and non-protein components of the HMW fraction

The HMW fraction was subjected to 100% ammonium sulphate precipitation to yield the protein (P-HMW) and non-protein components (NP-HMW). The protein component consisted of protein and large amount of carbohydrate, with a carbohydrate to protein ratio of 8. About 79.7% of the carbohydrate was alpha glucan and oligomers etc. and only 1.1% was beta glucan. On the other hand, the non-protein component (NP-HMW) was 99.3% carbohydrate, of which 57.5% was alpha glucan and oligomers etc. and 3.2% was beta glucan.

### Inhibition of TNF-alpha production

**Figure 2:**
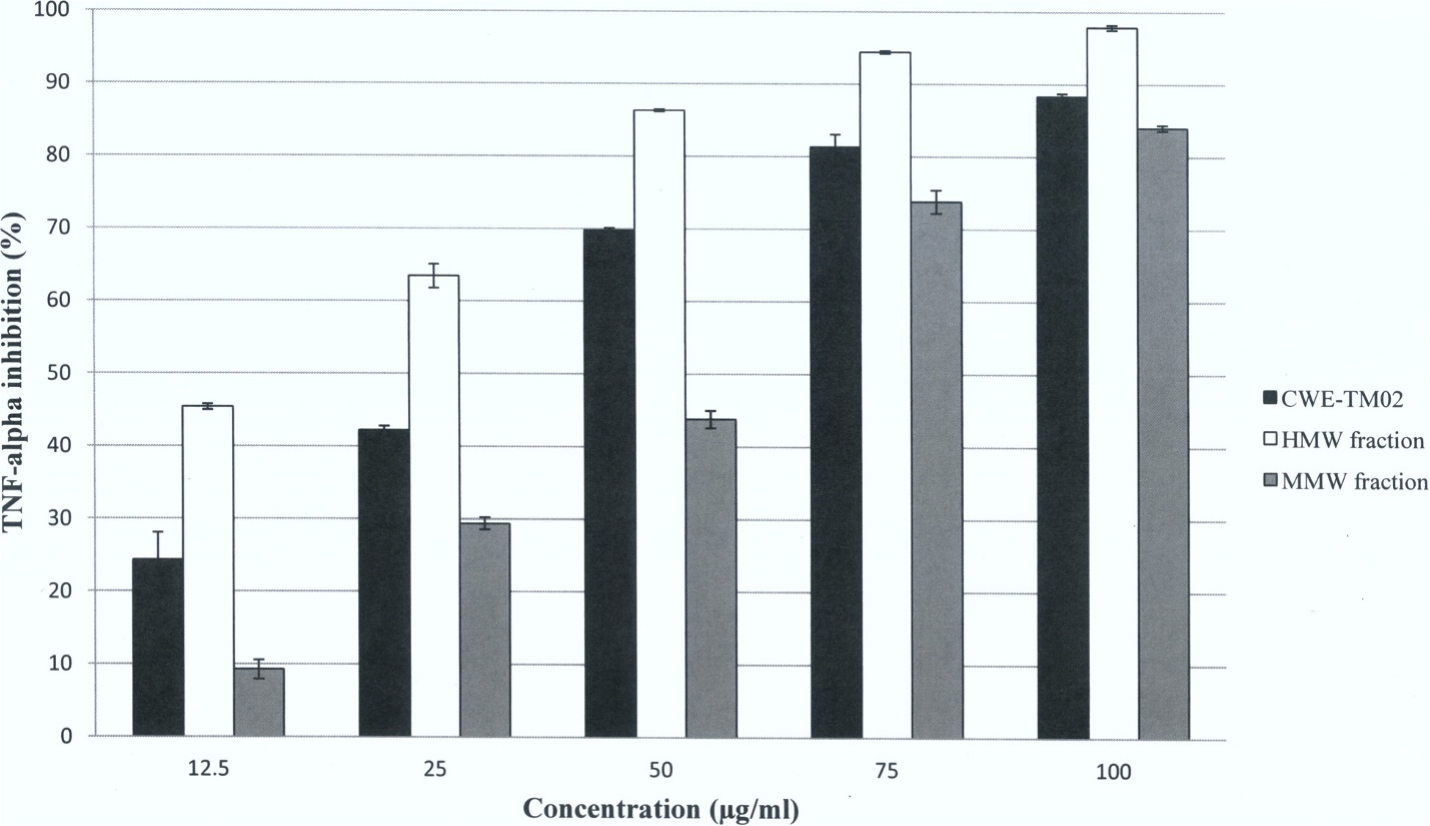
**The inhibitory effect of the cold water extract (CWE) and its HMW and MMW fractions on LPS-induced TNF-alpha production in RAW 264.7 cells.**

The CWE and its high and medium molecular weight fractions (HMW and MMW) all exhibited dose-dependent TNF-alpha inhibition activity (Figure 2, in all experiments, cell viability ≥ 75%). The half maximal inhibitory concentration (IC_50_) of CWE, HMW and MMW was 32.0, 16.0 and 56.0 μg/ml, respectively. The low molecular weight fraction did not exhibit the inhibitory activity. The protein component of the HMW exhibited very potent TNF alpha inhibition activity with IC_50_ of 0.76 μg protein/ml (data not shown), but the non-protein component did not exhibit any inhibitory activity.

## Discussion

The medicinal mushroom *L. rhinocerotis* has been used extensively by the indigenous people from Peninsular Malaysia. Usually, the sclerotium of the mushroom is grated on a hard surface with some water, and the resulting aqueous mixture is further diluted with water and consumed, with or without boiling [5, 15]. These methods of preparation mimic cold and hot water extract and hence cold and hot water extractions were used in the study in the investigation of anti-inflammatory activity of the mushroom. The methanol extraction was used to investigate if the non-polar, water insoluble substances also contribute to the anti-inflammatory activity.

Carrageenan induced rat hind paw edema has been widely used for evaluation of anti-inflammatory drugs as the relative potency estimates obtained from most drugs tend to reflect clinical experience [16]. Inflammation induced by carrageenan is acute, well researched and highly reproducible [17]. The course of carrageenan induced paw edema consisted of three phases [18]. The first phase is mediated by histamine and serotonin during the first hour, after which the increased vascular permeability is maintained by kinin (secondary phase) release up to 2.5 h [18]. From 2.5-6.0 h, the mediator appears to be a prostaglandin (final phase), release of which is closely associated with migration of leucocytes into the inflamed site [18].

The cold water extract exhibited anti-acute inflammatory activity by reducing paw edema induced by carrageenan at all the doses tested (25, 50, 100 and 200 mg/kg), during all three phases of edema development. Significant paw edema inhibition in a dose dependent manner was observed in rats treated with CWE from 25–200 mg/kg (*p* < 0.05) at 2^nd^ -5^th^ h post carrageenan injection. The anti-inflammatory pattern of CWE was similar to 10 mg/kg of indomethacin (an NSAID) in which all three phases of edema development induced by carrageenan were also inhibited. The CWE at 200 mg/kg showed greater extent of paw edema inhibition than that of 10 mg/kg indomethacin during all three phases of edema development. Lakshimi et al. [19] reported that ethanol extract of *Ganoderma lucidum* mycelium at 1000 mg/kg showed 65% paw edema inhibition after 3 h of carrageenan injection whereas CWE at lower dosage (200 mg/kg) gave 88% paw edema inhibition. Beta glucan rich extract from *Caripia montagnei* at 50 mg/kg significantly reduced the paw edema induced by carrageenan at 71% [20]. It should be noted, however, that the experimental design of the above mentioned findings (including ours) vary from one another and as such, it is not possible to make quantitative comparison.

In 200 mg/kg of cold water extract, the amount of the HMW, MMW and LMW fractions were 53 mg/kg, 35 and 112 mg/kg, respectively. Paw edema inhibition activity of the HMW fraction at 53 mg/kg dosage was comparable to that of 200 mg/kg CWE, whereas 35 mg/kg of MMW and 112 mg/kg of LMW fraction both showed substantially lower anti-inflammatory activity. This showed that the anti-inflammatory activity of CWE is mainly contributed by HMW fraction but the possibility of synergistic effect between HMW, MMW and LMW fractions cannot be excluded. CWE also exhibited *in vitro* anti-inflammatory activity as shown by its inhibitory effect on TNF alpha production in LPS-induced-macrophages, and both its HMW and MMW fractions exhibited strong inhibitory activity.

The nature of the active principle in the CWE causing anti-inflammatory activity has yet to be elucidated. A possible candidate is the polysaccharides-protein complexes or polysaccharides found in the extract. It has been reported that the anti-inflammatory properties of *Phellinus rhinosus* (Berk) and *Poria cocos* cultured mycelia were attributed to the polysaccharide-protein complex and water-soluble polysaccharide, respectively [21, 22]. The HMW fraction consists of 4% protein and 79% carbohydrates. The protein component isolated by ammonium sulfate precipitation, exhibited very potent TNF alpha inhibitory activity but the nature of the bioactive protein(s) that exhibit the anti-inflammatory activity in the protein component are yet to be elucidated. Since the protein component contained large amount of carbohydrate (8 parts of carbohydrate to 1 part of protein, mostly alpha glucan), the polysaccharide-protein complex may play a role in the anti-inflammatory effect. On the other hand, the non-protein component that was essentially carbohydrate (and mainly alpha glucan, with only 3.2% beta glucan) did not inhibit TNF-alpha production and hence was devoid of anti-inflammatory activity.

The hot water extract (HWE) and methanol extract (ME) at the dosage of 200 mg/kg were far less effective in reducing paw edema induced by carrageenan in all three phases of edema development when compared to CWE, at the same dosage. The extraction yield of CWE, HWE and ME were 20% (w/w), 37% (w/w) and 5% (w/w), respectively. The major anti-inflammatory principle(s) of the sclerotial powder of *L. rhinocerotis* cultivar TM02 are in the cold water extract, in particular, its high molecular weight fraction (HMW). The HWE exhibited weaker anti-inflammatory activity but because of its high yield it also contains substantial amount of anti-inflammatory principle. The yield of ME was so low that it contains only negligible amount of the bioactive substance.

Granulomatous inflammation is a focal chronic inflammatory response to tissue injury evoked by a poorly soluble substance characterised by the accumulation and proliferation of leukocytes, principally of the mononuclear type [23]. Cotton pellet induced granuloma test is widely used to assess transudative, exudative and proliferative phase of inflammation [24]. In this study, CWE at 200 mg/kg did not show reduction of wet and dry weight of implanted cotton pellet, indicating that it might not inhibit the transudative and proliferative phase of chronic inflammation.

## Conclusion

All three extracts of the sclerotial powder of *L. rhinocerotis* cultivar TM02 possess anti acute-inflammatory effect but most of the bioactive substance(s) appear to be extractable by cold water. The anti-inflammatory activity of the cold water extract (CWE) is mainly contributed by its high molecular weight (HMW) fraction, in particular its protein component, which was very potent in the inhibition of TNF-alpha production with an IC_50_ of 0.76 μg/ml. However, the possibility of synergistic effect between HMW, MMW and LMW fractions cannot be excluded. Cold water extract at 200 mg/kg did not inhibit transudative and proliferative phase of chronic inflammation.

## Abbreviations

CWE: Cold water extract
HWE: Hot water extract
ME: Methanol extract
HMW: High molecular weight fraction
MMW: Medium molecular weight fraction
LMW: Low molecular weight fraction
LPS: Lipopolysaccharide.
